# Retraction: *Mansonella*, including a Potential New Species, as Common Parasites in Children in Gabon

**DOI:** 10.1371/journal.pntd.0014178

**Published:** 2026-04-07

**Authors:** The PLOS Neglected Tropical Diseases Editors

The *PLOS Neglected Tropical Diseases* Editors retract this article [1,2] due to concerns about compliance with the PLOS Human Subjects Research policy.

The Materials and Methods section in [1] reports that the study involved blood samples obtained between 2011 and 2014 from febrile and afebrile children in Gabon, as well as blood samples previously collected in the Republics of Côte d’Ivoire and Senegal. The ethics approval statement reported in this article refers to agreements N°00370/MSP/CABMD and N°0023/2013/SG/CNE for the samples collected in Gabon, and N°86/MSLS/CNERN-dkn and N°00.87 MSP/DS/CNERS for the samples collected from the Republics of Côte d’Ivoire and Senegal respectively. PLOS notes that these ethics approval reference numbers have also been reported in multiple other publications (S1 File) despite there appearing to be major differences in the aims and objectives, populations, locations, methodologies, and types of samples described in these studies.

A representative of the Aix-Marseille Université Ethics Committee stated that the study complied with applicable legislation and ethical standards, and that citation of the same ethics approval number in several publications complies with international ethics standards when the opinion leads to several discoveries or lines of research justifying separate publications. The representative provided the following Ethics approval documents for editorial review:

- Ethics approval document N°0023/2013/SG/CNE, issued on 10 February 2013 by the Comité National d’Ethique pour la recherche of the République Gabonaise for a study titled “*Etude des pathogènes parasitaires et bactériens responsables des syndromes fébriles et digestifs chez les enfants vivant en Afrique Centrale*”
- Ethics approval document N°86/MSLS/CNERN-dkn, issued on 31 October 2014 by the Ministère de la Santé et de la Lutte contre le SIDA of the République de Cote d’Ivoire for a study titled “*Identification des agents pathogènes responsables de la fièvre dans la région de Yamoussoukro, République de Côte d’Ivoire*”
- Ethics approval document N°00.87 MSP/DS/CNERS, issued on 02 June 2010 by the Ministère de la Santé et de la Prévention of the République de Sénégal for a study titled “*Identification des agents pathogènes responsibles de fièvre au Sénégal (projet IDEPATH) dans les sites suivants: Niakhar (Fatick), Mlomp (Ziguinchor), Banafassi (Kédougou) et Keur Momar Sarr(Louga).*”

PLOS reviewed the documentation provided by the institution and concluded that the documents did not fully resolve the journal’s concerns. Specifically,

- Ethics approval document N°0023/2013/SG/CNE only partially covers the sample collection period reported for Gabon. In addition, PLOS noted that the president of the ethics board, who signed the ethics approval document, is also listed as a co-author on the article, raising concerns about the impartiality of the ethics assessment this study received.
- The second Gabonese ethics approval document reported in the article, N°00370/MSP/CABMD, was not provided for editorial review. In the absence of additional ethics approval documentation from the Republic of Gabon, PLOS is unable to confirm that independent, prospective ethics approval was obtained for the full sample collection period as would be required per PLOS’ policies for Human Subjects Research.
- The article does not report when the samples from Côte d’Ivoire and Senegal used in this study were collected. This information is needed to evaluate the article’s compliance with the PLOS Human Subjects Research policy.

JBLD and OM did not agree with the retraction. GM, FF, ANM, SMN, PBM, RZM, CHBE, FB, and DR either did not respond directly or could not be reached.

## Supporting information

S1 File List of other articles of which PLOS is aware that cite the same ethics approval reference number(s) reported in this study. (XLSX)

## References

[1] Mourembou G, Fenollar F, Lekana-Douki JB, Ndjoyi Mbiguino A, Maghendji Nzondo S, Matsiegui PB, et al. RETRACTED: *Mansonella*, including a Potential New Species, as Common Parasites in Children in Gabon. PLoS Negl Trop Dis. 2015;9(10):e0004155. doi: 10.1371/journal.pntd.0004155 26484866 PMC4618925

[2] The *PLOS Neglected Tropical Diseases* Editors. Expression of Concern: *Mansonella*, including a Potential New Species, as Common Parasites in Children in Gabon. PLoS Negl Trop Dis. 2022;16(12):e0010921. doi: 10.1371/journal.pntd.0010921 36512531 PMC9747013

